# Reduced Sexual Desire in Young Norwegian Women: A Mixed-Methods Study

**DOI:** 10.1007/s12119-022-09977-3

**Published:** 2022-06-01

**Authors:** Ashley Rebecca Bell, Ebba Giil, Bente Træen

**Affiliations:** Department of Psychology, University of Oslo, Blindern, P.O Box 1094, 0317 Oslo, Norway

**Keywords:** Reduced sexual interest, Sexual desire, Gender roles, Social expectations

## Abstract

Sexuality has become an area of social competence in which individuals strive to be recognized as sexually competent performers. However, a large proportion of young women experience reduced sexual desire. In this study, both quantitative and qualitative methods were applied. Using data from a questionnaire, the aim of this study was to explore the prevalence of, and the factors associated with, lack of sexual interest and desire among young women in Norway. Further, with the help of in-depth interviews, we investigated how young women with reduced sexual desire experience living with their desire problems and what they experience as the cause of their problems. The sample in the quantitative study consisted of 505 Norwegian women between the ages of 18 and 29 years. ANOVA was employed to explore differences in scores on psychological factors and relational factors, as well as between participants who experienced a lack of sexual interest and desire, and those who did not. The results indicate a high prevalence of lack of sexual interest and desire among women (37.1%), while low appearance satisfaction and low relationship satisfaction were central predictors of lack of sexual interest and desire. The sample in the qualitative study consisted of ten Norwegian women with reduced sexual desire between the ages of 18 and 29 years. Through thematic analysis, four themes emerged as experiences of living with reduced sexual desire: (1) physical and mental health, (2) being a young woman in today’s society, (3) relational factors, (4) negative experiences, personal expectations toward sexuality, and sexual trauma.

## Introduction

Sex is a universally experienced and desired human activity (Brotto, 2017). However, many women experience sexual problems through their lifetime (Mitchell et al., 2013; Peixoto & Nobre, 2015). Studies have found reduced sexual interest and desire to be the most common sexual problem reported among women, and it increases with age (Graham et al., 2020; Hendrickx et al., 2015; Kingsberg & Woodard, 2015). The limited research on reduced sexual interest and desire in younger women suggests that it is more widespread than what is commonly believed (Hendrickx et al., 2016; Mitchell et al., 2013). As per different studies, the reported prevalence of reduced sexual interest and desire among young women varies from 14 to 49% (Hendrickx et al., 2015; Mitchell et al., 2013; Richters et al., 2003; Štulhofer et al., 2011; Træen & Stigum, 2010), depending on how the concepts are defined and operationalized, the time period of the occurrence of the problem, and the studied samples. For example, in a British population-based study 25% of women aged between 16 and 24 years reported that they had experienced reduced sexual interest for a period of three months or longer in the last 12 months (Graham et al., 2017; Mitchell et al., 2013). Similarly, in a previous Norwegian study, 31% of women aged between 18 and 29 years reported reduced sexual desire at any time or most of the time during the past 12 months (Træen & Stigum, 2010), that made the authors claim that reduced sexual desire should be considered a public health problem. In the following section, we review the known predictors of reduced sexual interest and desire among women, with particular emphasis on young adult women, since most of the previous studies have largely focused on a varied age range from young to older women.

Research has found a relationship between low levels of education and sexual problems among adult women in general (Peixoto & Nobre, 2015). Low income may cause financial worries, causing stress, which can affect sexual desire (Štulhofer et al., 2011). Furthermore, sexual offences may affect sexual desire (Garneau-Fournier et al., 2017; Tuchik & Hassija, 2014), where feelings of shame may block sexual desire (Lorentzen et al., 2008; Træen & Sørensen, 2008). Negative thoughts also disconnect a person from the sexual moment (Carvalho & Nobre, 2010a, 2010b). For instance, a negative body image has been found to be associated with reduced sexual desire in young women (Seal et al., 2009). A negative body image may increase worries about what one’s body looks like during sex (Seal et al., 2009), which in turn may reduce sexual interest and desire. Previous research has also found that relational factors are important for women’s sexual interest and desire (Laan & Both, 2011; Mark et al., 2014). Research indicates a relationship between the duration of the relationship and reduced sexual desire in women, which may be explained by increased familiarity with the partner, monotony, and routine (Levine, 2002; Murray & Milhausen, 2012). Furthermore, research indicates that intimacy and emotional closeness are central factors for women’s sexual desire (Basson, 2000, 2002; Mark et al., 2014), and reduced sexual interest and desire may occur when women do not feel desired and accepted by their partners (Brotto et al., 2009). Additionally, differences in sexual desire between partners in a relationship have the potential to cause worries and negative emotions, which can further affect sexual satisfaction negatively (Sutherland et al., 2015). Therefore, it seems that emotional and psychological aspects of women’s relationships are important to women’s sexual desire.

Reduced sexual interest and desire may lead to sexual avoidance (Moor et al., 2021), and “duty sex” among women (Træen & Skogerbø, 2009). Also, young women are shown to continue to have sex despite feeling pain during intercourse (Elmerstig et al., 2013; Vannier & O’Sullivan, 2010). This could be related to an anxiety for not being perceived as a sexually competent person by the partner (Mark & Lasslo, 2018; Træen, 2008), hoping that desire will appear during sexual activity, or an attempt to avoid conflicts with the partner (Herbenick et al., 2014; Vannier & O’Sullivan, 2010). Young women with reduced sexual desire also often seem to blame themselves after having had sex they did not want (Bay-Cheng & Eliseo-Arras, 2008). At the same time, a desire to please the partner, prioritizing partners enjoyment before their own, living up to social and cultural expectations, and gender-specific norms are central themes in the literature on why young women have sex without desire (Braksmajer, 2017; Elmerstig et al., 2013; Vannier & O’Sullivan, 2010).

Considering the limited and contradicting findings of previous research on young women’s sexual interest and desire, and that women more often report experiencing reduced sexual interest and desire compared to men (Graham et al., 2017; Hendrickx et al., 2014), this study’s current aim is to explore this phenomenon among young Norwegian women. The existing literature suggests that social expectations, gender stereotypes, and gender roles continue to affect women’s sexuality. However, does this still apply to the younger generation of women in the Norwegian context, who are brought up with gender equality and a strong focus on women’s rights? To answer this question, more quantitative and qualitative research is needed, therefor mixed-methods was employed. In the quantitative sub-study, we explored the prevalence and predictors of lack of sexual interest, while the qualitative sub-study examined how young women experience living with reduced sexual desire, and what they think is the cause of their problems.

## The Quantitative Sub-Study

### Methods

Data for the quantitative sub-study were retrieved from an Internet survey conducted in March 2020 by the University of Oslo, in cooperation with Kantar. Participants were recruited through Kantar’s Gallup Web Panel (https://www.galluppanelet.no). Everyone in the Gallup Web Panel is randomly recruited, without the possibility of self-recruitment. Participants who were randomly drawn to participate in the study was assured of anonymity and informed that participation was voluntarily, following ethical guidelines developed for market- and poll organization surveys. A total of 11,685 persons registered in Kantar’s Gallup Web Panel were randomly contacted and asked to participate in an online study on sexuality. Of the invited panel members, 4160 individuals participated in the study (response rate = 35.6%), with the majority filling out the questionnaire on a mobile phone (51%). For a more detailed description of the study, we referred to the existing publications from the study (Fischer & Træen, 2021).

Eligibility for the current study was determined by gender, including only female participants between the ages of 18 and 29 years (N = 505). The selected age range was chosen based on previous research’ using this, or similar age ranges, to address young women (Mitchell et al., 2013; Træen & Stigum, 2010). The average age of the female subsample was 24.9 years. One of four women (24.2%) had a masters’ level education, 40.8% had a bachelor’s level education, and 35.0% had further lower levels of education. The majority of the women (71.7%) reported no religious affiliation, 22.0% were Christians, 3.0% were Muslims (15 women), and 17 women did not respond to the question. Even though most Norwegians are registered in the Norwegian church, most Norwegians do not consider themselves as having a religious affiliation (Urstad, 2017). Hence, the high percentage of women not having a religious affiliation in our sample is consistent with the general younger population. The sample consisted mostly of heterosexual women (86.1%), while only one in ten identified themselves as lesbian, bisexual or pansexual (9.5%). Most of the women had partners (62.8%), while 37.0% were not in a relationship.

### Measures

#### Outcome variable

The dependent variable *Reduced sexual interest or desire* was measured by asking participants if they had experienced a lack of interest in having sex over a period of three months or longer in the last 12 months. The response options were 0 = no and 1 = yes. The question was retrieved from National Survey of Sexual Attitudes and Lifestyles, NATSAL-3, and the DSM-5 definition of reduced sexual interest (Mitchell et al., 2016). The term “sexual interest” is often used interchangeably with the term “sexual desire” (Öberg et al., 2004; Peixoto & Nobre, 2015; Štulhofer et al., 2011). In the Diagnostic and Statistical Manual of Mental Disorders (DSM-5), both the terms “sexual interest” and “sexual desire” are used while describing clinical sexual dysfunction (American Psychiatric Association, 2013). This corresponds to research that has found a strong association between the two conditions sexual interest and sexual desire (Laumann et al., 1999). In this present study, the terms “sexual interest” and “sexual desire” will be used interchangeably.

#### Predictor variables

*Level of education* was assessed by asking, “What is your highest level of formal education?" The response categories were 1 = primary school (6–8 years at school), 2 = lower secondary school (9–10 years at school), 3 = higher secondary school or high school (12–13 years at school), 4 = college or lower university level (bachelor’s degree level or similar), and 5 = higher university level (master’s degree, Ph.D. level or similar).

*Relationship status* was assessed by asking: “What is your marital status?” The response options were 1 = unmarried, 2 = separated/divorced, 3 = widow/widower, and 4 = married/cohabitant/registered partnership. Participants who responded that they were unmarried, separated/divorced or widow/widower, were additionally asked, “Are you currently in a permanent relationship?” The response options were 1 = no, 2 = yes, with one person, and 3 = yes, with several people.

*Appearance satisfaction* was measured with the question: “How dissatisfied or satisfied are you with your physical appearance?” The response options were evaluated on a 7-point scale ranging from 1 = very dissatisfied to 7 = very satisfied.

*Satisfaction with weight* was measured with the question: “How dissatisfied or satisfied are you with your weight?” with answer options ranging from 1 = very dissatisfied to 7 = very satisfied.

*Sexual activity satisfaction* was measured with the question: “In general, how satisfied are you with your current level of sexual activity?” The response options ranged from 1 = very satisfied to 5 = very unsatisfied.

*Satisfaction with the current relationship* was measured by asking: “All things considered, how satisfied are you with your current relationship?” The response options were evaluated on a 7-point scale ranging from 1 = not satisfied at all to 7 = completely satisfied.

*Sexual avoidance* was measured by the question: "Have you, during the last 12 months, deliberately avoided having sex with your partner?" with answer options ranging from 1 = never to 5 = very often.

### Statistical Analysis

SPSS 26.0 was used to perform all data analysis. Analysis of variance (ANOVA) was used to examine whether there were differences in scores on psychological and cohabitation factors between the participants who experienced a lack of sexual interest and those who did not experience a lack of sexual interest. Furthermore, logistic regression analysis was performed to examine the likelihood of a lack of sexual interest based on selected predictor variables. The selected predictor variables used in the analyses were education level, relationship status, appearance satisfaction, satisfaction with weight, sexual satisfaction, satisfaction with current relationship, and frequency of sexual avoidance.

### Results

A total of 37.1% of women reported lack of sexual interest. More women with a partner (41.4%) than without a partner (27.7%) reported a lack of sexual interest (Pearson’s Chi-square 7.54, 1 d.f., *p* = 0.006). More overweight women (43.8%) reported a lack of sexual interest (Pearson’s Chi-square 5.21, 1 d.f., *p* = 0.022) as compared to women who were not overweight or were underweight (32.4%). No other statistically significant differences were found between sociodemographic factors and the lack of sexual interest.

To investigate differences in lack of sexual interest, psychological factors, and relationship factors, an ANOVA analysis was conducted (see Table 1).

**Table 1:** Satisfaction with selected aspects of oneself and frequency of sexual avoidance among 18–29-year-old Norwegian women, who have and do not have sexual interest (Mean and Standard Deviation (St.D.))

	N	Mean	St.D.	F	Sign.
Appearance satisfaction *(1 = very dissatisfied, 7 = very satisfied)*				9.84	.002
Have sexual interest	278	4.73	1.26		
Lacked sexual interest	164	4.34	1.30		
Satisfaction with weight *(1 = very dissatisfied, 7 = very satisfied)*				3.12	.078
Have sexual interest	275	3.95	1.54		
Lacked sexual interest	164	3.68	1.60		
Sexual activity satisfaction *(1 = very satisfied, 5 = very unsatisfied)*				11.68	.001
Have sexual interest	271	3.46	1.18		
Lacked sexual interest	160	3.06	1.12		
Satisfaction with current relationship *(1 = not satisfied at all, 7 = completely satisfied)*				10.14	.002
Have sexual interest	180	6.34	0.87		
Lacked sexual interest	126	6.01	0.98		
Frequency of sexual avoidance *(1 = never, 5 = very often)*				71.70	.000
Have sexual interest	176	1.80	0.81		
Lacked sexual interest	124	2.71	1.04		

Women who lacked sexual interest were less satisfied with their relationship, with their own appearance, and avoided sex more often than women who did not lack sexual interest. Women who lacked sexual interest were also less satisfied with their level of sexual activity as compared to women who did not lack sexual interest.

### Model 1: All Women

The relationship between lack of sexual interest and sociodemographic factors, psychological factors, and cohabitation factors was studied using two logistic regression models. Model 1 included all the women in the study, and in Model 2, only women who had a permanent current partner were included (see Table 2).

**Table 2:** Lacking sexual interest by a selected sample of predictors related to social background, psychological and relationship factors among 18–29-year-old Norwegian women (multiple logistic regression analysis)

	%	Model 1 (All women, n = 384)		Model 2 (Partnered women, n = 255)	
		AOR	95% CI	AOR	95% CI
*Education level*					
Lower	42.0	1.00		1.00	
Higher	34.9	0.69^ ns^	0.43–1.11	1.13^ ns^	0.57–2.23
*Relationship status*					
Not in a relationship	27.7	1.00		1.00	
In a relationship	41.4	2.12^**^	1.33–3.63	–	–
*BMI*					
Underweight and normal weight	32.4	1.00			
Overweight	43.8	1.73^*^	1.03–2.88	1.69^ ns^	0.82–2.46
*Appearance satisfaction*					
(with a unit’s increase)		0.68^**^	0.53–0.87	0.74^ ns^	0.53–1.05
*Satisfaction with weight*					
(with a unit’s increase)		1.16^ ns^	0.94–1.43	1.17^ ns^	0.88–1.54
*Satisfaction with current relationship*					
(with a unit’s increase)		–	–	0.88^ ns^	0.62–1.25
*Sexual activity satisfaction*					
(with a unit’s increase)		–	–	0.68^**^	0.51–0.91
*Frequency of avoiding sex*					
(with a unit’s increase)		–	–	2.48^***^	1.78–3.47

The first column shows bivariate analyzes, percentage experiencing lacked sexual interest. The second column shows multivariate logistic regression analysis with adjusted odds ratio (AOR) with 95% confidence intervals (CI) for reporting lacked sexual interest

^***^p < .001; ^**^p < .01; ^*^p < .05; ^ns^ = not statistical significant different

As shown in Table 2, several predictor variables were significantly correlated with the outcome variable. Women who were in a relationship were more likely to report lack of sexual interest than those who were not in a relationship (AOR = 2.12, *p* < 0.01). Furthermore, women who were overweight were more likely to report a lack of sexual interest (AOR = 1.73, *p* < 0.05). Appearance satisfaction was also a significant factor in the lack of sexual interest (AOR = 0.68, *p* < 0.01). No other variables included in Model 1 had a statistically significant association with lack of sexual interest.

### Model 2: Women with Partners

Compared to women who were satisfied with their level of sexual activity, dissatisfied women were more likely to report a lack of sexual interest (AOR = 0.68, *p* < 0.01). Similarly, compared to women who reported not avoiding sex, women who avoided sex were more likely to report lacking sexual interest (AOR = 2.48, *p* < 0.001). None of the other variables included in Model 2 had a statistically significant association with lack of sexual interest, which indicates that relationship factors were more important predictors of lack of sexual interest than sociodemographic factors.

### Discussion

This study showed that over one in three young Norwegian women lacked interest in having sex. Women with a permanent partner reported lack of sexual interest more often than single women. Women who actively avoided having sex also reported lack of sexual interest more often than women who did not. Being overweight, dissatisfaction with the level of sexual activity, and appearance, also affected sexual interest.

The results showed a slight increase from previous Norwegian studies regarding lack of sexual interest and desire in women (Træen & Stigum, 2010). Relationship factors were found to be more important than sociodemographic factors in predicting lack of sexual interest in women, which has also been found in previous studies (Dewitte & Mayer, 2018; Mark et al., 2014). Lack of sexual interest may negatively affect the relationship in that the problem leads to an imbalance within the relationship. Research indicates that different levels of sexual desire between two individuals in a relationship can cause anxiety and distress, and affect their sexual and relationship satisfaction (Mark, 2012; Sutherland et al., 2015; Willoughby & Vitas, 2012). On the contrary, relationship satisfaction may negatively affect women’s sexual interests (Ridley et al., 2006). The length of the relationship and dynamics between the two people in the relationship seems to be crucial for women's sexual desire and interest (Dewitte & Mayer, 2018; Murray & Milhausen, 2012). In other words, the longer the relationships have lasted, and the more negative dynamics there are between the people in the romantic relationship, the more negative influence the relationship has on young women’s sexual interest.

All participants who reported a lack of sexual interest also avoided sex more often, which previous research has also found for women of all ages (Hendrickx et al., 2016). Moor et al. (2021) found that women with impaired sexual desire often avoided all physical contact with their partner in order to prevent unwanted sexual activity (Moor et al., 2021). Furthermore, the women in this study who were not satisfied with the sexual activity in the relationship, reported a lack of sexual interest more often. This is in line with our findings, which showed that low satisfaction with sexual activity was associated with a lack of sexual interest in women.

Satisfaction with one’s own appearance was also related to a lack of sexual interest in young women. Previous research has shown similar results; having good self-esteem about one's own body is related to sexual desire (Seal et al., 2009). The reason why single women with lower satisfaction with appearance experienced a lack of sexual interest may be related to objectification and portrayal of women in society. Constantly monitoring one’s own body can lead to increased body shame, triggering negative emotions related to one’s sexuality, which in turn can contribute to sexual problems (Fredrickson et al., 1998). In this way, it can be assumed that objectification and portrayal of women in society affect women’s sexual interests and desires. Furthermore, the results showed that women who were overweight lacked sexual interest more often than women who were not overweight or were underweight. Although being overweight has not been found to be a risk factor for sexual problems in young women in the past (Brody, 2004), Seal et al. (2009) reported that weight related to physical traits was particularly related to appetite problems in women. In light of the often-unrealistic body ideals women face today (Murnen & Don, 2012), being overweight and deviating from the ideals can lead to a poorer body image, that may result in a lack of sexual interest and desire in women.

Some limitations of the current study need to be addressed. It is important to note that there was a general low response rate to experiencing lack of sexual interest in the quantitative sub-study. This may be a result of challenges in reporting whether one experiences a lack of sexual interest, as sexual problems are considered taboo to talk about (Træen, 2008). Additionally, only one question was used to examine whether participants had lack of sexual interest. Even though participants who reported it, received one follow-up question; future research should include more questions to explore young women’s sexual interests and desires. Likewise, future research should include questions on both the terms sexual desire and sexual interest to see if there is a correlation between the terms among young women.

This survey showed that a significant proportion of young Norwegian women lacked sexual interest. The variables considered in the study point to some explanations for the possible causes. However, qualitative data may give us further insight into the causes and mechanisms underlying the lack of sexual interest among young women.

## The Qualitative Sub-Study

### Methods

Ten women with reduced sexual desire aged 18 to 29 years were recruited to participate in an in-depth interview study about how they understood the causes of their sexual problems. The participants were recruited through an online poster, published on the researchers’ own Facebook and Instagram profiles. Thereafter, women who showed an interest in participation contacted the researchers through e-mail or telephone. Data were collected through interviews, following a semi-structured interview guide. All interviews lasted between 40 and 120 min and were conducted in December 2020. Due to the ongoing Covid-19-pandemic, all but one interview was conducted using the University of Oslo Zoom tool through the researchers own University of Oslo user accounts. The advantages of video-based interviews are that they can be conducted anywhere, which proved helpful because we had participants from varied geographical regions in Norway (Deakin & Wakefield, 2014; Hanna, 2012). The transcripts were written verbatim immediately after the interviews were conducted. Neither the recordings nor the transcribed interviews could identify the participants, and fictitious names were used to ensure anonymity. Thematic analysis was performed to identify repeated patterns of causes for reduced sexual desire and the experience of living with it. Both inductive and deductive approaches were applied, although the deductive approach dominated. Both researchers coded the entire dataset separately to obtain a broader comprehension and better interpretation of data. Subsequently, we went through all the codes together and themed them. The quotations presented in this study have been translated from Norwegian to English, in addition to being edited to achieve greater readability.

### Results and Discussion

Most of the participants said that they were social persons, and that they viewed themselves as dutiful by either working, studying, or participating in voluntary duties. The participants were also concerned with the norms and expectations as women. Most women expressed that they had a feminist view of gender norms, although they expressed fairly gender stereotypical attitudes throughout the interview. We identified four different themes related to the causes of reduced sexual desire and the subsequent experience of living with reduced sexual desire. The themes were: (1) physical and mental health, (2) being a young woman in today’s society, (3) relationship factors, and (4) negative experiences, personal expectations toward sexuality, and sexual trauma.

### Physical and Mental Health

Painful sex was a physical ailment experienced by several participants. This is in line with previous research, that indicates that approximately every third woman between the ages of 16 and 29 experiences painful sex (Christensen et al., 2011; Richters et al., 2003). A significant correlation has been found between reduced sexual interest and desire, and pain during sex in adult women in general (Mitchell et al., 2016; Peixoto & Nobre, 2015). Moreover, it is important to note that painful sex can be both a cause of, and a consequence of, reduced sexual desire. Many women described that they had sex without feeling the desire prior to sex, or that their desire disappeared during sex. When Victoria (25 years) was asked if she had had sex when the desire was not present or disappeared, she replied, *“Yes, many times. What can I say, what happens is probably that the desire passes very quickly, and I don’*t *quite understand why?”* This finding is consistent with previous research that has shown that many young women participate in sexual activities despite sexual problems or lack of sexual desire (Quinn-Nilas & Kennett, 2018). Why do young women have sex without desire, especially if they experience pain? The answer is often complex.

Most interviewees claimed that they had sex hoping that the desire would eventually appear. Some participants mentioned that they stopped sexual activity when the desire disappeared, but most of them continued having sex despite the lack of desire. This is in line with previous research (Vannier & O’Sullivan, 2010). Pain as a consequence of having sex without desire was expressed mostly in interviewees who were in a romantic relationship, which is likely as these women had access to a partner who expected sex regularly. Recalling the time with her latest boyfriend, Hannah (27 years) said, “*I chose to have sex several times without being quite ready, and it was often very painful.*”

Many participants said they used hormonal contraceptives, antidepressants, and other medications, or that they had previously been diagnosed with a mental disorder. However, none of the participants considered these factors in isolation, were the cause of their sexual desire problem, though they may have been contributing factors; has also been found in previous research among women (Graham et al., 2017; Malmborg et al., 2016). Furthermore, many women said they felt exhausted, were sometimes in a bad mood, and that they prioritized other things in their life over sex. It should be noted that since the interviews took place during Covid-19 lock-down in Norway, participants may have been affected in varying degrees from increased stress, increased uncertainty, more isolation, and reduced social contact. Due to the pandemic, Julia's (24 years) life situation changed, with increased stress and concerns, that she believed resulted in her reduced sexual desire:

*I know some people use sex when they’re stressed. I think it’s the opposite for me, I need to be in a good state of mind to be able to have sex. And lately I have not been that, so yes, that effects [my desire]’.*

Even though most of the interviewees did not experience negative ripple effects of the pandemic to the same extent as Julia, stress and external influences were clearly contributing factors to reduced desire for many participants. These findings are consistent with previous research that found sexual desire to be affected by stress among young women (Vannier & O’Sullivan, 2010). Additionally, both disturbing and irrational thoughts embossed many participants, which may have influenced their sexual desire. These thoughts may appear before, during, or after sex. Anna had experienced disturbing thoughts several times: “*During foreplay or while having sex, I sort of think ‘Oh, now I must not think about what I’m having for dinner*.’ *And then I lay there thinking, oh, what should I have for dinner.”* This corresponds to Nobre’s (2009) finding that impaired sexual desire is related to several cognitive factors among women. Based on what the women said, it seems that disturbing thoughts can be both a cause and a consequence of living with reduced sexual desire.

### Being a Young Woman in Today’s Society

The next theme captures the interviewees’ experiences of what it is like to be a young woman in today’s society, and how this affected their sexual desire. The interviewees said that they perceived women’s and men’s sexuality to be different, and stereotypical gender role perceptions in society affected them. For example, they expressed a belief that men in general have a higher sex drive than women. Hannah explained: “*Of what I read in the media and maybe what I talk to my friends about is similar, I experience that maybe boys’ sex drive is higher than girls.”* In line with previous research, it emerged that to assent to general stereotypical gender role perceptions about sexuality in today’s society had a negative impact on women’s desire problems (Graham et al., 2017). Further, there was also a perception among the interviewees that as women, they had to suppress their sexuality to conform to the society’s expectations. This has also been validated in previous research with women between 28 and 76 years of age. (McCabe, Tanner, et al., 2010). Western culture, in general, is more accepting of men’s sexual activity, and women often receive social feedback about their sexual activity being risky and in disaccord with social norms (Cherkasskaya & Rosario, 2019). Anna recounted how violating stereotypical gender norms can give women negative social feedback: *“If women sleep around, they are seen as really loose or hookers, right? Women should not take initiative because that is wrong [according to society]. Then men feel a little less like men, it makes a crack in their manhood*.” Since female sexuality is socially less acceptable, the participants might have felt that they had to suppress their sexuality, including their sexual desire.

Furthermore, the interviewees talked about a feeling of body shame during sex, that may have stemmed from today’s media that is characterized by objectifying and sexualizing the presentation of women. This objectification and sexualization can lead to increased body shame, negatively impacting women’s sexual desire, where they are not able to relax and enjoy the sexual moment (Fredrickson et al., 1998). The findings of our quantitative sub-study thus conform to the previous study.

Apart from the experiences of body shame, the interviewees experienced pressure to perform sexually. For some women, their lack of desire was connected to performance anxiety and insecurity around their own sexual competence. Today’s society is characterized by having to perform in several social arenas, including sexual arenas. According to Lyttkens (1989), we are living in the age of social competence, where being a sexually competent individual is emphasized. Media, today, is filled with information about how to improve oneself as a sexual individual (Træen, 2008). Several interviewees stated that a negative mindset about expectations of one’s own sexual performance could have had an impact on their sexual desires. Furthermore, many participants experienced a form of performance anxiety during sexual activity, which could have led to their focusing more on how they should perform sexually. Regarding this, Nora (25 years) said:*“I get high performance anxiety sometimes, and I believe it affects my desire. I get so stressed, ‘like now, I [feel] have to get horny.’ I just get stressed instead.”*

Based on Lyttkens’s (1989) theory, revealing one’s problems with sexual desire can make one feel sexually incompetent; further leading to women hiding their problems and continuing to have sex without desire. Many participants mentioned that having sex without desire was a strategy they used to prove to themselves and their partner(s) that they were sexually competent individuals. Norwegian society is characterized by openness about sexuality and a focus on sexual skills, that may act as a template for women’s assessment of their own sexual competence. The interviewees felt that they did not live up to their own and other people’s expectations of being sexually competent. “*Maybe there is something wrong with me*,” said Victoria when she was asked about what triggers the awareness of her reduced sexual desire. A feeling of deviating from what is considered normal may have led many of the interviewees to hide their lack of desire from others.

It appeared that the interviewees not only experienced appearance and sexual pressure, but also a pressure to perform on different social arenas in life (Lyttkens, 1989). Cutting off sexual desire could be a way to handle the pressures in social arenas.

### Relationship Factors

As shown in the quantitative sub-study, relationship factors played a significant role in reduced sexual desire among many participants. Previous research (Murray & Milhausen, 2012) also confirms that the longer women had been in the relationship, the more their sexual desire decreased. With time, relationships also move from a stage of novelty where everything is new, to a stage with more knowledge about the partner where monotony and routine take over (Basson, 2000; Levine, 2002). Considering that our interviewees are young women, most of them may not have been in their relationships long enough to experience this. Instead, poor couple dynamics, breach of trust, unfulfilled need for tenderness and emotional support may be some of the reasons for reduced desire to have sex. Mary (25 years) talked about how she previously had a boyfriend who did not respect and support her the way she needed, and this affected her sexual desire: “*I noticed that I was not being appreciated, not being valued, and I did not feel good and safe.*” Furthermore, clinical research has shown that sexual problems can be a result of dysfunctional or dissatisfying relationships (McCabe, Althof, et al., 2010). This may have been a reason for the lack of sexual desire for some interviewees who had experienced relationships characterized by a lack of respect, tenderness, and support. In line with Mark et al.’s (2014) research, many of the interviewees said that they valued the emotional aspects of their relationships, and that these aspects were important for their sexual desire.

Most of the interviewees believed that sexually satisfying their partners was important. Some of the women claimed that satisfying their partner made them happy, even if they did not experience sexual desire and pleasure. Sara (25 years) had recently got into a relationship and talked about how she had sex just to satisfy her boyfriend: "*It’s a bit often; I only have sex to finish it and satisfy him. I don’t do it for my own pleasure*.” The purpose of this kind of sexual activity is often to satisfy the partner's needs or to preserve the committed relationship (Elmerstig et al., 2013). Some researchers claim that such self-sacrifice is characteristic of romantic relationships and that, in some cases, a consequence of this strategy might be increased intimacy in the relationship (Impett et al., 2015). On the contrary, in some cases the division between self-sacrificing acts and sexual coercion may become more blurred. Women in general are often expected to put their partner’s needs before their own (Strazdins & Broom, 2004). The fact that many women repeatedly engage in “duty sex” (Træen & Skogerbø, 2009), may have a detrimental or adverse effect, both on the relationship and on the individual’s sexual desire. Although previous research has not explored this among young women to a great extent, our findings show that young women also believe that it is important to satisfy their partner sexually.

Many interviewees felt that sexual activity was expected when in a relationship, or they felt pressured to have sex with their partner, which reduced their sexual desire. These sexual expectations were linked to concerns about hurting or making the partner feel uncomfortable by saying “no” to sex. Research shows that having sex to avoid a partner’s bad mood or not to hurt him, often leads to poorer relationship quality and lowers sexual satisfaction in both partners (Impett et al., 2005; Muise et al., 2013). A few of the participants also said that when they did not want sex, they felt that their partner would be whining or accusing them of being asexual or that there was something wrong with them. To not be perceived as sexually incompetent, many interviewees had sex without desire. Maintaining sexual activity in the relationship can therefore function as a strategy that women use to maintain an image of themselves as a sexually competent individual, both for the partner and themselves (Lyttkens, 1989; Træen, 2008).

### Negative Experiences, Personal Expectations Toward Sexuality, and Sexual Trauma

Based on the interviewees’ narratives, previous negative sexual experiences and low personal expectations of their own sexuality may have contributed to reduced sexual desire. Most of the women said that they had experienced several negative and unsatisfactory sexual encounters in their past. This is in line with previous research, which found that many women have experienced sexual encounters, both with committed and casual partners, as disappointing, frustrating, and sexually dissatisfying (Leiblum, 2002). Mary explained that she had not received sexual satisfaction from several casual partners: “*I have had many one-night-stands, and they rarely give me anything. If you don’t get an orgasm, it’s just a penetration. Most of the time I just fake it because I don’t think it’s satisfying*.” What happens when one repeatedly experiences unsatisfactory sexual encounters?

Interpreting the interviewees’ narratives, it is likely that it further reduces one’s sexual desire. The women also said that previous negative sexual experiences reduced their desire to have sex with new partners. As observed by Schnarch (1997), many women must experience a type of sex that feels worth having to want sex. Furthermore, it has been found that women are likely to attribute responsibility and mistakes to themselves (Maass & Volpato, 1989). This may, in turn, produce a feeling of being sexually incompetent or that there is something wrong with oneself (Lyttkens, 1989; Træen, 2008). Baumeister (2000) claims that women have higher erotic plasticity than men. This can lead to increased vulnerability to external influences and compel women to act in ways that are not in their own best interests. This may explain why many of the interviewees had experienced negative sexual experiences and had few expectations of their own sexual satisfaction.

Most of the interviewees had experienced different types of trauma. Some had experienced the death of a loved one and believed that this contributed to their reduced sexual desire. This finding has also been reported in previous studies on men and women exposed to trauma (De Silva, 1999). Moreover, half of the women had experienced sexual assault; two interviewees had experienced it in their childhood. These experiences contributed to their reduced sexual desire, which is in accordance with previous research (Garneau-Fournier et al., 2017). In the study, women’s reduced sexual desire may be a result of negative associations with sexual activity attached to the aversive sexual experience (O’Loughlin & Brotto, 2020). Moreover, it has been found that experiencing trauma in childhood is associated with sexual problems later in adulthood for women of all ages (O’Loughlin et al., 2020). Victoria was among the women who had experienced several traumatic events. Having experienced a sexual relationship at the age of 6 and rape at the age of 23, she expressed a strong distrust in men and was afraid of being exploited for others’ hedonistic needs. She explained that her reduced sexual desire could be a result of her sexual traumas in the past: “*I don’t know if there’s a kind of defense mechanism that I have. Sex for me is very vulnerable. (…) I have been sexually exploited; therefore, sex is vulnerable. It probably weakened my desire a lot*.”

Guilt and shame were often something the participants were affected by, after being subjected to abuse. Malin elaborated:

*I was diagnosed with PTSD [after the rape] (...) I had bruises from top to bottom, and for a long time I had problems realizing that it was a rape [that had happened] (…) I felt that it [the rape] was my fault, since I had actually joined him into that room*.

Shame has been shown to inhibit sexual desire for victims of sexual assault (Lorentzen et al., 2008; Træen & Sørensen, 2008). Sofia had experienced several traumas, including sexual abuse, miscarriage, and an aggressive boyfriend. She considered the combination of these events to be the cause of her reduced sexual desire. Sofia expressed an inherent fear of experiencing the painful feelings again that were associated with the traumatic events, particularly since she was ashamed of the traumas on losing control of her own body for a long time: “*I didn’t want to have sex afterwards for a long, long time. I did not want to masturbate either, I was ashamed for a long time.*’ Even though many interviewees talked about a feeling of shame or guilt related to their trauma, this was not the case for everyone. Nora experienced being gang raped when she was 16 years old, before her first intercourse. After the rape, she became hypersexual and remained so for a long period of time, which was later followed by reduced sexual desire. Nora’s experience of becoming hypersexual as a result of sexual rape is in line with previous research, which indicates that such sexual behavior is distinctive in some rape victims (Slavin et al., 2020). Though participants talked about different kinds of sexual abuse, the common feature was it affected women negatively. Many of them had experienced sexual aversion, and many said the abuse had contributed to their reduced sexual desire, which is in line with previous research (Garneau-Fournier et al., 2017; Turchik & Hassija, 2014).

It is important to reflect on the limitations of this qualitative study. Those who participated in the study may have been more willing to share their experiences of having reduced sexual desire than others. Another limitation of the sub-study was that it did not ask or consider the participants’ sexual orientation, which may have had implications for the study, such as making incorrect assumptions about their sexual orientation. Though, some interviewees talked about their sexual orientation on their own initiative; their sexual identity did not seem to be related to reduced sexual desire. Lastly, it is important to note that this study was conducted during Covid-19, which may have had unforeseen influence on the research process and the interviews.

## Conclusion

In the quantitative sub-study, we found a high prevalence of reduced sexual interest and desire among 18–29-year-old Norwegian women. In accordance with Træen and Stigum’s (2010) study a decade ago, this study indicates that reduced sexual interest and desire is still considered a public health problem among young Norwegian women. Quantitative and qualitative data supported each other in that appearance satisfaction and relationship factors were important for experiencing sexual interest and desire. The qualitative sub-study suggests that the mechanisms underlying reduced sexual interest and desire in young women may be varied and complex. Both sub-studies indicate that sexual interest and desire in young women is affected by the time-period we live in, where social and sexual competence are highly valued. The qualitative study indicates that many women still have a repressive role in sexual contexts; perhaps most visible in victims of sexual trauma and abuse, and one of the long-term effects is reduced sexual desire. Most women considered living with reduced sexual interest and desire as a problem, but few did not. Nevertheless, all interviewees claimed that reduced sexual desire had affected their experience of their own sexuality. More attention needs to be devoted to this problem by health professionals and researchers.

## Data Availability

The data for the two studies are currently available only to researchers invited to participate in the research team.
